# Intake of ultra-processed foods and asthenozoospermia odds: A hospital-based case-control study

**DOI:** 10.3389/fnut.2022.941745

**Published:** 2022-10-20

**Authors:** Jia-Le Lv, Qi-Jun Wu, Xiao-Bin Wang, Qiang Du, Fang-Hua Liu, Ren-Hao Guo, Xu Leng, Bo-Chen Pan, Yu-Hong Zhao

**Affiliations:** ^1^Department of Clinical Epidemiology, Shengjing Hospital of China Medical University, Shenyang, China; ^2^Clinical Research Center, Shengjing Hospital of China Medical University, Shenyang, China; ^3^Liaoning Key Laboratory of Precision Medical Research on Major Chronic Disease, Shengjing Hospital of China Medical University, Shenyang, China; ^4^Department of Obstetrics and Gynecology, Shengjing Hospital of China Medical University, Shenyang, China; ^5^Center of Reproductive Medicine, Shengjing Hospital of China Medical University, Shenyang, China

**Keywords:** asthenozoospermia, case-control study, diet, ultra-processed foods, China

## Abstract

**Background:**

The intake of ultra-processed foods (UPFs) has increased rapidly in recent years. Evidence has suggested that UPFs has adverse effects on several health outcomes. This study aimed to first evaluate the association between the intake of UPFs and asthenozoospermia odds.

**Methods:**

A hospital-based case-control study including 549 cases and 581 controls was performed in the infertility clinics of Shengjing Hospital of China Medical University from June 2020 to December 2020. Dietary intake was assessed using a validated food frequency questionnaire. Food items were categorized by the NOVA classification system based on the degree of processing. Semen parameters were analyzed according to the World Health Organization guidelines.

**Results:**

The highest tertile of UPFs intake (% of total energy intake) was positively associated with the odds of asthenozoospermia (odds ratio [OR] = 1.53; 95% confidence interval [CI]: 1.12, 2.10; *P* for trend < 0.05), compared with the lowest tertile. Similar patterns were also found in subgroup analyses among participants with age ≥32 years (OR = 1.58; 95% CI: 1.04, 2.40), BMI ≥ 24 kg/m^2^ (OR = 1.52; 95% CI: 1.04, 2.22), ever cigarette smoking (OR = 1.78; 95% CI: 1.14, 2.79), and ever alcohol drinking (OR = 1.65; 95% CI: 1.01, 2.72), and in sensitivity analyses by using absolute amount (g/day) to calculate the intake of UPFs.

**Conclusion:**

Higher consumption of UPFs was positively associated with the odds of asthenozoospermia. More studies are needed to confirm our findings.

## Introduction

Infertility is a major issue of human reproductive health, affecting approximately 15% of couples worldwide (1). Half of all infertility cases are linked to male factors (1, 2). Asthenozoospermia, manifested as decreased sperm motility, is a major pathological indicator of male infertility (3, 4). An epidemiological study from China suggested that the prevalence of asthenozoospermia showed an upward trend during 2008–2016, and it could be more than 50% among 38,905 infertile male patients (5). Although the etiology of asthenozoospermia remains incompletely understood (6), several genetic and environmental factors have been reported to contribute to the risk of asthenozoospermia (7, 8). For example, a DNAH17 missense variant induces flagella destabilization and consequently causes asthenozoospermia (6), and tobacco smoking is associated with decreased sperm motility by impairing mitochondrial function in the sperm tail region (9).

As one of the most important environmental factors, the diet factor has also been proposed to play crucial roles in the etiology of asthenozoospermiais (10, 11). Ultra-processed foods (UPFs) are formulations of ingredients, mostly of exclusive industrial use, that result from a sequence of industrial processes (12), and they have been indicated to have many adverse effects on human health (13, 14). UPFs are often overconsumed due to their presentation, marketing, and the characteristics of highly palatable, attractive, and convenient. The contribution of UPFs can be more than 50% of total daily energy in some high-income countries, such as Britain and the United States (15, 16). Although the consumption of UPFs in China is relatively low, it is increasing rapidly in recent years (17). UPFs are commonly high in added sugar, total fat, saturated fat, and low in protein, dietary fiber, and micronutrients (18, 19), and evidence has suggested that UPFs can create suitable environment for microbes in the gut, then promote some inflammatory disease, such as obesity and metabolic syndrome (20). Notably, inflammation may be the major cause of the development of asthenozoospermia (21). In addition, a case-control study with 107 incident asthenozoospermic men and 235 age-matched controls suggested that the western diet pattern, characterized by high intake of UPFs, was positively associated with asthenozoospermia (odds ratio [OR] = 2.86; 95% confidence interval [CI]: 1.83, 2.97) (22). Thus, the above evidence hints that the intake of UPFs may have an adverse impact on asthenozoospermia.

To the best of our knowledge, there has been no efforts to explore the relationship between the intake of UPF and the odds of asthenozoospermia. Therefore, the purpose of this study was to investigate the potential association of aforementioned topic.

## Materials and methods

### Study design and participants

This study followed the Strengthening the Reporting of Observational Studies in Epidemiology (STROBE) checklist (Supplemental Table 1). The details of the study design and participants have been described in previously published studies (3, 11). Briefly, a hospital-based case-control study was performed at Shengjing Hospital of China Medical University, in Liaoning, China. Participants were recruited in the infertility clinics from June, 2020 to December, 2020.

**Figure 1:**
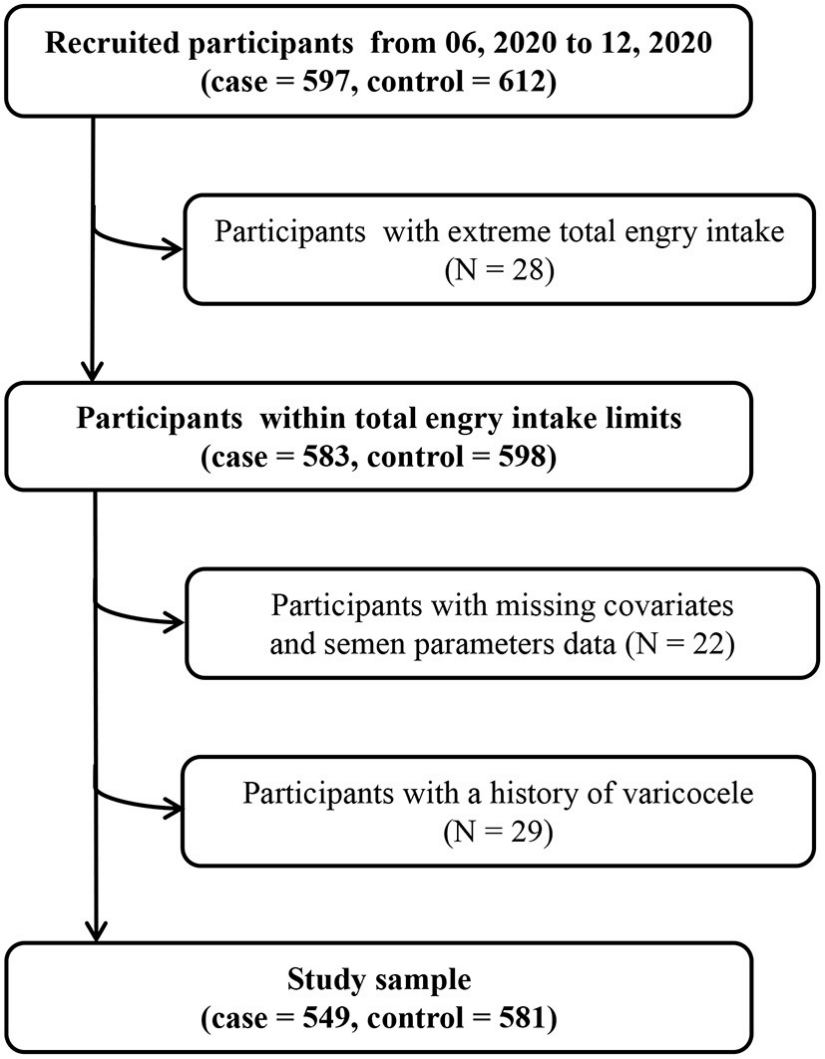
Flowchart of study participants.

Asthenozoospermia cases (*n* = 597) were diagnosed according to the fifth edition of World Health Organization laboratory manual for the examination and processing of human semen, and defined as total motility (progressive + non-progressive) <40%, including both rapidly and slowly progressive motility, sluggish motility, and non-progressive motility (23). In addition, asthenozoospermia was also defined as progressive motility <32%, including rapidly and slowly progressive motility, and sluggish motility in the same class within 60 min of ejaculation over the past 3 months (23). The total number (or concentration) of spermatozoa and percentage of morphologically normal spermatozoa was equal to or above lower reference limits (24). Eligible controls (*n* = 612) were normozoospermic men from infertile couples, with ≥15 × 10^6^ of sperms/mL, ≥40% total motility, ≥32% progressive motility, and ≥4% normal forms (11). According to the sample size calculation method of the unmatched case-control study design, we assumed that OR = 1.5, α = 0.05, β = 0.2, and the proportion of UPF intake in the control group was 67%, the PASS 11.0 software was used to calculate the sample size. The result showed that 469 participants were needed for both case and control groups. We included participants who consented to participate and returned the completed study questionnaire, and we excluded participants with total energy intake outside of predefined limits (total energy intake <800 or >4,200 kcal/d) (*N* = 28), missing covariates and semen parameters data (*N* = 22), and a history of varicocele (*N* = 29). Finally, 549 cases and 581 controls were included in our study and met the requirement of sample size. In addition, the participation rates of cases and controls were 91.96 and 94.93%, respectively (Figure 1). This study was approved by the ethics committee of Shengjing Hospital of China Medical University (No. 2017PS190K).

### Semen collection and analysis

Semen samples were obtained by masturbation into a plastic tube in a dedicated semen collection room after 3–7 days abstinence periods. Condoms or lubricants were not allowed to be used. The samples were allowed to be liquefied for 45–60 min before analysis. Ejaculate volume was directly measured, and other semen parameters were measured using the WLJY9000 instrument, such as sperm concentration, total sperm count, motility, and morphology. Sperm DNA fragmentation and high sperm DNA staining were evaluated using BriCyte E6 flow cytometry. Pasteurization was applied for sperm smear observations and the sperm morphology was evaluated using optical microscope. All analyses were carried out by an experienced technician, and an external quality control was carried out throughout the study.

### Data collection

Face-to-face interviews were performed by trained professional interviewers using a validated questionnaire. Demographic and lifestyle factors were collected, including age, annual family income (<50, 50 to <100, or ≥100 RMB thousand yuan), educational level (junior secondary or below, senior high school, technical secondary school, or junior college/university or above), dietary intake, dietary change status (yes or no), ever cigarette smoking (yes or no), ever alcohol drinking (yes or no), and physical activity. For dietary change status, participants were asked whether they had changed their diets since this year and before. Cigarette smoking and alcohol drinking were defined as at least one per day and once a week, respectively, for more than six consecutive months. In addition, physical activity was evaluated according to the previous study (25). Briefly, participants were asked the duration of activities spent on work, transportation, housework, and leisure-time exercise in the past 12 months (25), and the metabolic equivalent of tasks (MET) from the 2011 update of a major compendium of physical activities was used to calculate the amount of physical activity (26). Furthermore, physical examination data, such as height and weight, were also measured by trained health professionals. Body mass index (BMI) was used as a noninvasive proxy measure of body fatness and calculated as weight in kilograms divided by the square of the height in meters (kg/m^2^) (27). According to the cut-off point for Chinese, normal/underweight and overweight/obesity were defined as BMI ≤23.9 and ≥24 kg/m^2^, respectively (28).

### Dietary assessment

A validated semi-quantitative food frequency questionnaire (FFQ) which included 110 food items with specified serving sizes was used to assess dietary intake for participants over the past year. The FFQ used in the present study was based on FFQ used in a large cohort study in the northeast of China (29, 30). The reproducibility and validity of the FFQ used in the current study were similar to FFQ used in previously published literature (11, 30–32). The FFQ included seven frequency categories (“never,” “two to three times per month,” “one time per week,” “two to three times per week,” “four to six times per week,” “one time per day,” and “more than two times per day”) for each food item. Daily consumption for each food item was calculated by multiplying the specified portion size by the frequency at which each food item was consumed per day. Daily intake of nutrients was calculated according to the Chinese Food Composition Table (33), and calculated by multiplying daily consumption for each food item by its nutrient content per gram and then adding the nutrient contributions of all food items.

To assess the overall nutritional quality, principal component analysis was used to generate major dietary patterns and factor loadings for all food items except UPF foods in grams (34). The suitability of data for factor analysis was examined using the Kaiser-Meyer-Olkin (KMO) and Bartlett's test of sphericity (35). The KMO value was 0.905 and Bartlett's test of sphericity was significant (*P* < 0.001), suggesting that data was suitable for factor analysis. Varimax rotation was used to increase data interpretability (30). After evaluating eigenvalues (>1) and conducting the screen test, five factors were identified. The factors were named descriptively according to the food groups showing high factor loadings (absolute value ≥0.3) in each dietary pattern (36), including vegetables pattern, fruit pattern, fish pattern, processed foods pattern, and tea pattern (Supplemental Table 2).

### Exposure assessment

All food items were classified by the NOVA classification system, which categorizes foods into four groups according to the nature, extent, and purpose of food processing (37). Two investigators (J-LL and F-HL) independently classified food items according to previous studies (12, 19), and any discrepancies in this process were resolved by reaching a consensus with the third investigator (Q-JW). The definitions of the four food groups and the classification of food items of the FFQ were shown in Supplemental Table 3. Our study typically focused on the UPFs group, including instant noodles, bread, sausages, preserved egg, western-style pastry or cakes, Chinese pastries or cakes, cookies, sweet snacks or candies or jams, chocolate, ice cream, sesame/soybean paste, vegetable/fruit drinks, and soft drinks. The proportion of UPFs weighted by total energy intake (% total energy intake) for each participant was calculated.

### Statistical analysis

The Kolmogorov-Smirnov test was used to assess the normality of continuous variables. To assess the characteristics of participants, continuous variables were all presented as median and interquartile range as they were not normally distributed, and categorical variables were presented as number and percentage. To compare differences between groups, the Kruskal-Wallis was used for continuous variables, and the Chi-square test was used for categorical variables. The proportion of UPFs weighted by total energy intake (% total energy intake) was categorized into three groups according to control distribution with the lowest tertile as the reference group. In the main analysis, Unconditional logistic regression analyses were used to assess associations between UPFs and the odds of asthenozoospermia. ORs and 95% CIs were calculated. The covariates included in the model were determined by univariate logistic regression and published literature for asthenozoospermia (3, 11, 24, 38). Model 1 was adjusted for age (continuous, years), BMI (continuous, kg/m^2^), and total energy intake (continuous, kcal). Model 2 was further adjusted for annual family income (< 50, 50 to <100, or ≥ 100 RMB thousand yuan), educational level (junior secondary or below, senior high school, technical secondary school, or junior college/university or above), ever cigarette smoking (yes or no), ever alcohol drinking (yes or no), abstinence time (continuous, days), physical activity (continuous, MET/h/week), and dietary change status (yes or no) based on Model 1. To consider the potential effect of nutritional quality, we further adjusted these five dietary patterns (continuous) in Model 3 based on Model 2. There was no collinearity among all variables in our analysis. Linear trends across tertiles were tested by modeling the median value of each tertile as a continuous variable.

Subgroup analyses were performed to examine potential effect modifications according to age (<32 or ≥32 years), BMI (<24 or ≥24 kg/m^2^), ever cigarette smoking (Yes or No), and ever alcohol drinking (Yes or No). And the likelihood test was used to evaluate potential interactions between UPFs intake and stratification variables.

We additionally evaluated the association between absolute amount of UPFs intake (g/day) and the odds of asthenozoospermia in sensitivity analyses to examine the robustness of the results. In the secondary analysis, we also assessed the effects of unprocessed or minimally processed foods and processed foods on asthenozoospermia. All statistical analyses were performed with SAS software version 9.4 (SAS Institute Inc., Cary, NC, USA). All statistical tests were two sided and statistical significance was set at *P* < 0.05.

## Results

The distribution of general characteristics among cases and controls is shown in Table 1. Compared with controls, cases had significantly (*P* < 0.05) higher age and abstinence time, but lower proportion of ever alcohol drinking and semen parameters (sperm concentration, total sperm count, progress motility, total motility, and normal sperm morphology). In terms of diet and related nutrients intake, cases had significantly (*P* < 0.05) higher consumption of sodium than controls.

**Table 1:** General characteristics of participants.

**Characteristics**	**Case**	**Control**	***P* value**
Participants (*n*)	549	581	
Age (years), median (IQR)	33.00 (30.00–36.00)	32.00 (29.00–34.00)	< 0.05
BMI (kg/m^2^), median (IQR)	26.17 (23.72–28.73)	25.95 (23.36–28.73)	0.36
Physical activity (MET/h/week), median (IQR)	132.83 (100.70–217.17)	127.57 (98.35–226.67)	0.69
Television watching (hours/week), median (IQR)	3.00 (0.00–10.00)	3.00 (0.00–10.00)	0.53
Computer using (hours/week), median (IQR)	21.00 (14.00–30.00)	21.00 (14.00–30.00)	0.56
Abstinence time (days), median (IQR)	4.00 (3.00–5.00)	4.00 (3.00–5.00)	< 0.05
**Educational level (** ***n*** **, %)**			0.67
Junior secondary or below	121 (22.04)	141 (24.27)	
Senior high school/ technical secondary school	79 (14.39)	82 (14.11)	
Junior college/university or above	349(63.57)	358(61.62)	
**Annual family income (RMB thousand yuan) (** ***n*** **, %)**			0.76
< 50	98 (17.85)	94 (16.18)	
50– < 100	209 (38.07)	226 (38.90)	
≥100	242 (44.08)	261 (44.92)	
**Cigarette smoking (** ***n*** **, %)**			0.11
Yes	264 (48.09)	307 (52.84)	
No	285 (51.91)	274 (47.16)	
**Alcohol drinking (** ***n*** **, %)**			< 0.05
Yes	199 (36.25)	250 (43.03)	
No	350 (63.75)	331 (56.97)	
**Dietary change before anticipation (** ***n*** **, %)**			0.17
Yes	127 (23.13)	115 (19.79)	
No	422 (76.87)	466 (80.21)	
**Semen parameters**, median (IQR)			
Ejaculate volume (ml)	3.40 (2.5–4.40)	3.20 (2.50–4.00)	0.12
Sperm concentration (10^6^/ml)	49.13 (33.01–73.44)	62.26 (42.63–87.59)	< 0.05
Total sperm count (10^6^/ml)	169.20 (108.4–255.42)	211.18 (131–297.53)	< 0.05
Progress motility (%)	23.18 (15.93–28.41)	43.06 (37.85–50.40)	< 0.05
Total motility (%)	29.06 (20.77–35.38)	53.65 (46.44–62.23)	< 0.05
Normal sperm morphology (%)	5.00 (4.00–7.00)	6.00 (4.00–8.00)	< 0.05
**Diet and related nutrients intake**, median (IQR)			
Energy (kcal/day)	1,731.23 (1,401.51–2,149.24)	1,683.75 (1,400.51–2,048.60)	0.12
Proteins (% energy)	16.49 (15.12–17.77)	16.46 (15.17–17.78)	0.79
Fat (% energy)	24.99 (21.68–27.95)	24.73 (22.06–27.98)	0.74
Carbohydrates (% energy)	57.29 (52.76–62.23)	57.27 (52.09–61.39)	0.08
Total dietary fiber (g/1,000 kcal)	8.99 (7.45–10.92)	8.74 (7.23–10.65)	0.07
Sodium (mg/1,000 kcal)	637.83 (547.96–754.00)	602.39 (505.25–723.28)	< 0.05

BMI, body mass index; MET, metabolic equivalent of task; IQR, interquartile range.

All continuous variables were shown as the mean and standard deviation. P values were determined with the Kruskal-Wallis or chi-square test. All statistical tests are two sided.

Table 2 displays the main associations between the intake of UPFs and the odds of asthenozoospermia. We found that the highest tertile of UPFs intake was positively associated with the odds of asthenozoospermia in model 1 (OR = 1.46; 95% CI: 1.08, 1.98; *P* for trend < 0.05) and model 2 (OR = 1.48; 95% CI: 1.09, 2.00; *P* for trend < 0.05), compared with the lowest tertile. And this positive association remained significant in the fully adjusted model 3 (OR = 1.53; 95% CI: 1.12, 2.10; *P* for trend < 0.05).

**Table 2:** Adjusted odds ratio (95% confidence interval) of ultra-processed foods intake and asthenozoospermia.

**Models**	**Tertiles of**			***P* for**
	**UPFs intake**			**trend**
	**T1**	**T2**	**T3**	
Range (% of total energy intake)	< 8.71	8.71– ≤ 15.12	>15.12	
Case/Control	163/194	167/193	219/194	
Model 1	1.00 (Ref)	1.32 (0.98, 1.79)	1.46 (1.08, 1.98)	< 0.05
Model 2	1.00 (Ref)	1.09 (0.81, 1.48)	1.48 (1.09, 2.00)	< 0.05
Model 3	1.00 (Ref)	1.11 (0.82, 1.51)	1.53 (1.12, 2.10)	< 0.05

T, tertile; Ref, reference; UPF, ultra-processed foods.

Model 1: adjusted for age, body mass index, and total energy intake.

Model 2: adjusted for age, annual family income, educational level, body mass index, cigarette smoking, alcohol drinking, abstinence time, physical activity, total energy intake, and dietary change status.

Model 3: adjusted for age, annual family income, educational level, body mass index, cigarette smoking, alcohol drinking, abstinence time, physical activity, total energy intake, dietary change status, and dietary patterns except UPF foods (vegetables pattern, fruit pattern, fish pattern, processed foods pattern, and tea pattern).

Table 3 presents the results of subgroup analyses. We observed that the intake of UPFs showed an association with a higher odds of asthenozoospermia among participants with age ≥ 32 years (OR = 1.58; 95% CI: 1.04, 2.40), BMI ≥ 24 kg/m^2^ (OR = 1.52; 95% CI: 1.04, 2.22), ever cigarette smoking (OR = 1.78; 95% CI: 1.14, 2.79), and ever alcohol drinking (OR = 1.65; 95% CI: 1.01, 2.72). However, no multiplicative interactions were found between UPFs intake and these stratifying variables (all *P* for interaction > 0.05).

**Table 3:** Adjusted odds ratio (95% confidence interval) of ultra-processed foods intake and asthenozoospermia stratified by age, BMI, cigarette smoking, and alcohol drinking.

**Characteristics**	**Case**	**Control**	**Tertiles of UPFs intake (% of total energy intake)**			***P* for interaction**
			**T1**	**T2**	**T3**	
**Age (years)**						0.74
< 32	221	276	1.00 (Ref)	1.12 (0.71, 1.77)	1.29 (0.80, 2.08)	
≥32	328	305	1.00 (Ref)	1.55 (1.03, 2.35)	1.58 (1.04, 2.40)	
**BMI (kg/m** ^**2**^ **)**						0.39
< 24	149	176	1.00 (Ref)	1.95 (1.10, 3.51)	1.86 (1.02, 3.43)	
≥24	400	405	1.00 (Ref)	0.94 (0.65, 1.36)	1.52 (1.04, 2.22)	
**Cigarette smoking (** ***n*** **)**						0.51
Yes	264	307	1.00 (Ref)	1.16 (0.74, 1.82)	1.78 (1.14, 2.79)	
No	285	274	1.00 (Ref)	1.12 (0.72, 1.73)	1.23 (0.78, 1.96)	
**Alcohol drinking (** ***n*** **)**						0.58
Yes	199	250	1.00 (Ref)	1.27 (0.77, 2.10)	1.65 (1.01, 2.72)	
No	350	331	1.00 (Ref)	1.11 (0.75, 1.66)	1.50 (0.99, 2.28)	

BMI, body mass index; T, tertiles; Ref, reference; UPF, ultra-processed foods.

Adjusted for age, annual family income, educational level, body mass index, cigarette smoking, alcohol drinking, abstinence time, physical activity, total energy intake, dietary change status, and dietary patterns except UPF foods (vegetables pattern, fruit pattern, fish pattern, processed foods pattern, and tea pattern).

In the fully adjusted model (model 3), the positive association was also observed when evaluating the association between absolute amount of UPFs intake (g/day) and the odds of asthenozoospermia (Supplemental Table 4). In addition, we found that unprocessed or minimally processed foods intake was negatively associated with the odds of asthenozoospermia, and processed foods intake was positively associated with the odds of asthenozoospermia (Supplemental Table 5).

## Discussion

In this hospital-based case-control study, to our knowledge, we first found that the highest tertile of UPFs intake was positively associated with the odds of asthenozoospermia, compared with the lowest tertile. Similar patterns were also observed among participants with age ≥ 32 yeas, BMI ≥ 24 kg/m^2^, ever cigarette smoking, and ever alcohol drinking in the subgroup analyses, and in sensitivity analyses by using absolute amount to calculate UPFs intake.

Although no epidemiological study assessed the association between UPFs intake and the odds of asthenozoospermia, some previous observational studies regarding diet factors and the odds of asthenozoospermia have been in line with our findings (22, 24, 39). Indeed, Eslamian et al. suggested that the higher intake of total saturated fatty acids (OR = 1.85; 95% CI: 1.83, 2.97) and total trans-fatty acids (OR = 2.53; 95% CI: 1.54, 3.92) was positively associated with the odds of asthenozoospermia (24), and the western dietary pattern could increase the odds of asthenozoospermia (OR = 2.86; 95% CI: 1.83, 2.97) (22). Of note, UPFs are characterized by being rich in total saturated fatty acids and trans-fatty acids, and are also representative foods of western dietary pattern. In addition, Eslamian et al. reported that the intakes of some specific UPFs were associated with a higher odds of asthenozoospermia. Indeed, the odds of asthenozoospermia significantly increased in the highest tertile of processed meat (OR = 2.03; 95% CI: 1.70, 2.44) and sweets intake (OR = 2.05, 95% CI: 1.09, 2.26) (39).

Although the underlying mechanism is not yet clear, there have been some possible explanations for the positive association between UPFs intake and the odds of asthenozoospermia. First, UPFs strongly affect the nutritional quality of diets (40), and are rich in energy, fat, and added sugar (40, 41). Evidence suggested that poorer nutritional quality of UPFs may be partly contribute to weight gain (42), and the consumption of energy, fat, and sugar might increase the risk of overweight and obesity (43). Meanwhile, evidence from a large cohort study with 10,665 patients consulting for a semen analysis demonstrated that increased BMI had an adverse effect on semen quality (44), and the result of a label-free quantitative LC-MS/MS proteomic analysis indicated that declines in endoplasmic reticulum protein 57 (ERp57) and actin-binding-related protein T2 (ACTRT2) expression may play critical roles in reducing sperm motility, thereby contributing to obesity-induced asthenozoospermia (45).

Second, UPFs usually contain food additives, and recent studies had indicated that food additives could cause microbiota-mediated adverse effects in the host (20). In fact, emulsifiers can alter microbiota composition and increase its proinflammatory potential, thereby promoting the development of chronic inflammatory diseases (46, 47). Interestingly, a prospective study demonstrated that the dysbiosis of gut microbiota was associated with male infertility (48), and an animal study found that the dysbiosis of gut microbiota was one of the main causes for the impaired spermatogenesis and sperm motility (49). Notably, inflammation seems to play an important role in the etiology of asthenozoospermia (50), and TNF-a as a central regulator of inflammation had been reported significantly higher in asthenozoospermia patients compared with health controls (51). Hence, food additives contained in UPFs may promote inflammatory disorders by causing the dysbiosis of gut microbiota, thereby increasing the risk of asthenozoospermia. At present, food additives are used widely; however, there is still a lack of data on the long-term effects of them on human health, and the potential effects are still largely unknown (42). Therefore, more research on the relationship between food additives and asthenozoospermia is needed in the future.

Third, some contaminants can be formed during the industrial process. Indeed phthalates can migrate from plastic packaging to foods, and act as endocrine disruptors (52). An epidemiological study suggested that there was a positive association between UPFs intake and urinary concentrations of phthalate metabolites (53). And an animal study suggested that mice prenatally exposed to Di-(2-ethylhexyl) phthalate (one of the most used phthalates) displayed a wide range of gonadal and epididymal abnormalities, including asthenozoospermia (54).

### Strengths and limitations

Our study had some strengths. First, we provided a new sight for exploring the relationship between dietary factors and asthenozoospermia by considering the degree of food processing. Our findings supported the need for public health policies to limit the intake of ultra-processed foods and increase the intake of unprocessed or minimally processed foods instead. Second, our study had a relatively large sample size and high participation rates, which were conducive to reducing random errors. Third, our study adjusted for a variety of confounding factors to increase the credibility of our conclusions. However, our study also had some limitations. First, our FFQ was not designed by considering the NOVA classification, therefore the degree of processing of several food items was ambiguous. However, it is essential to reasonably recognize food items according to the NOVA classification. In order to reduce the bias caused by misclassification, the food items included in our FFQ were classified by two investigators independently, and any discrepancy was judged by the third investigator. Second, although FFQ was commonly used for dietary assessment, dietary intake of participants could not be calculated accurately based on the standard food portions and relatively limited food items number. Therefore, the measurement bias was unavoidable, and this might have led to the misestimation of the association between UPFs and asthenozoospermia. To reduce the bias, we excluded the participants who reported total energy intake values outside of predefined limits. Third, as a case-control study, selection biases and recall biases could not be avoided, and causation could not be confirmed either. To improve the comparability of case and control, the same clinic controls were applied, and highly trained and skilled researchers were selected to collect information for reducing the recall bias (55, 56). Fourth, although we had made the extensive adjustment, we still could not entirely exclude unmeasured or residual confounding factors, such as genetic factors and air pollution (57, 58). In addition, some important information, such as drug use history, was not considered because they were not cleaned. Last, our study was conducted in Chinese people, therefore the extrapolation of our findings to other populations was limited.

In conclusion, our study indicated that there was a positive association between the intake of UPFs and the odds of asthenozoospermia. Large prospective studies in different populations and settings are needed in the future to confirm our findings.

## Data availability statement

The raw data supporting the conclusions of this article will be made available by the authors, further inquiries can be directed to the corresponding authors.

## Ethics statement

The studies involving human participants were reviewed and approved by the Ethics Committee of Shengjing Hospital of China Medical University. The patients/participants provided their written informed consent to participate in this study.

## Author contributions

J-LL, Q-JW, X-BW, B-CP, and Y-HZ conceived the study. Q-JW, B-CP, and Y-HZ contributed to the design. X-BW, QD, R-HG, XL, and B-CP collected the data. JL-L and F-HL cleaned the data, checked the discrepancy, and analyzed the data. JL-L, Q-JW, X-BW, and B-CP interpreted the data. All authors interpreted the data, read the manuscript, and approved the final vision.

## Funding

This work was supported by the National Key R&D Program of China (No. 2017YFC0907403 to Y-HZ), Shengjing Hospital Clinical Research Project (No. M0071 to B-CP), 345 Talent Project of Shengjing Hospital of China Medical University (Q-JW), the Liaoning Revitalization Talents Program (No. XLYC1802095 to Y-HZ), and the unveiling and leading project of Liaoning province (No. 2021JH1/10400050 to Y-HZ).

## Conflict of interest

The authors declare that the research was conducted in the absence of any commercial or financial relationships that could be construed as a potential conflict of interest.

## Publisher's note

All claims expressed in this article are solely those of the authors and do not necessarily represent those of their affiliated organizations, or those of the publisher, the editors and the reviewers. Any product that may be evaluated in this article, or claim that may be made by its manufacturer, is not guaranteed or endorsed by the publisher.

## References

[1] Jiao SY Yang YH Chen SR. Molecular genetics of infertility: loss-of-function mutations in humans and corresponding knockout/mutated mice. Hum Reprod Update. (2021) 27:154–89. 10.1093/humupd/dmaa034 33118031

[2] Castaneda JM Hua R Miyata H Oji A Guo Y Cheng Y. TCTE1 is a conserved component of the dynein regulatory complex and is required for motility and metabolism in mouse spermatozoa. Proc Natl Acad Sci U S A. (2017) 114:E5370–8. 10.1073/pnas.1621279114 28630322 PMC5502601

[3] Liu FH Wang XB Wen ZY Wang HY Zhang M Zhang S. Dietary Inflammatory index and risk of asthenozoospermia: a hospital-based case-controlled study in China. Front Nutr. (2021) 8:706869. 10.3389/fnut.2021.706869 34395499 PMC8357981

[4] Dong FN Amiri-Yekta A Martinez G Saut A Tek J Stouvenel L. Absence of CFAP69 causes male infertility due to multiple morphological abnormalities of the flagella in human and mouse. Am J Hum Genet. (2018) 102:636–48. 10.1016/j.ajhg.2018.03.007 29606301 PMC5985338

[5] Wu ZG Chen WK Fei QJ Liu YL Liu XD Huang H. Analysis of semen quality of 38 905 infertile male patients during 2008-2016 in Wenzhou, China. Asian J Androl. (2021) 23:314–8. 10.4103/aja.aja_83_20 33433531 PMC8152416

[6] Zhang B Ma H Khan T Ma A Li T Zhang H. A DNAH17 missense variant causes flagella destabilization and asthenozoospermia. J Exp Med. (2020) 217:e20182365. 10.1084/jem.20182365 31658987 PMC7041708

[7] Jin ZR Fang D Liu BH Cai J Tang WH Jiang H. Roles of CatSper channels in the pathogenesis of asthenozoospermia and the therapeutic effects of acupuncture-like treatment on asthenozoospermia. Theranostics. (2021) 11:2822–44. 10.7150/thno.51869 33456575 PMC7806476

[8] Li L Chen S. Screening, identification and interaction analysis of key MicroRNAs and genes in Asthenozoospermia. Int J Med Sci. (2021) 18:1670–9. 10.7150/ijms.54460 33746583 PMC7976570

[9] Asare-Anane H Bannison SB Ofori EK Ateko RO Bawah AT Amanquah SD. Tobacco smoking is associated with decreased semen quality. Reprod Health. (2016) 13:90. 10.1186/s12978-016-0207-z 27496053 PMC4974764

[10] Eslamian G Amirjannati N Rashidkhani B Sadeghi MR Hekmatdoost A. Nutrient patterns and asthenozoospermia: a case-control study. Andrologia. (2017) 49:12624. 10.1111/and.12624 27246740

[11] Wang XB Wu QJ Liu FH Zhang S Wang HY Guo RH. The Association between dairy product consumption and asthenozoospermia risk: a hospital-based case-control study. Front Nutr. (2021) 8:714291. 10.3389/fnut.2021.714291 34746202 PMC8566545

[12] Monteiro CA Cannon G Levy RB Moubarac JC Louzada ML Rauber F. Ultra-processed foods: what they are and how to identify them. Public Health Nutr. (2019) 22:936–41. 10.1017/S1368980018003762 30744710 PMC10260459

[13] Fiolet T Srour B Sellem L Kesse-Guyot E Alles B Mejean C. Consumption of ultra-processed foods and cancer risk: results from NutriNet-Sante prospective cohort. BMJ. (2018) 360:k322. 10.1136/bmj.k322 29444771 PMC5811844

[14] Silva Meneguelli T Viana Hinkelmann J Hermsdorff HHM Zulet MA Martinez JA Bressan J. Food consumption by degree of processing and cardiometabolic risk: a systematic review. Int J Food Sci Nutr. (2020) 71:678–92. 10.1080/09637486.2020.1725961 32053758

[15] Monteiro CA Moubarac JC Levy RB Canella DS Louzada M Cannon G. Household availability of ultra-processed foods and obesity in nineteen European countries. Public Health Nutr. (2018) 21:18–26. 10.1017/S1368980017001379 28714422 PMC10260838

[16] Poti JM Mendez MA Ng SW Popkin BM. Is the degree of food processing and convenience linked with the nutritional quality of foods purchased by US households? Am J Clin Nutr. (2015) 101:1251–62. 10.3945/ajcn.114.100925 25948666 PMC4441809

[17] Baker P Machado P Santos T Sievert K Backholer K Hadjikakou M. Ultra-processed foods and the nutrition transition: Global, regional and national trends, food systems transformations and political economy drivers. Obes Rev. (2020) 21:e13126. 10.1111/obr.13126 32761763

[18] Monteiro CA Cannon G Moubarac JC Levy RB Louzada MLC Jaime PC. The UN Decade of Nutrition, the NOVA food classification and the trouble with ultra-processing. Public Health Nutr. (2018) 21:5–17. 10.1017/S1368980017000234 28322183 PMC10261019

[19] Zhang S Gan S Zhang Q Liu L Meng G Yao Z. Ultra-processed food consumption and the risk of non-alcoholic fatty liver disease in the Tianjin Chronic Low-grade Systemic Inflammation and Health Cohort Study. Int J Epidemiol. (2021) 51:237–49. 10.1093/ije/dyab174 34528679

[20] Zinocker MK Lindseth IA. The western diet-microbiome-host interaction and its role in metabolic disease. Nutrients. (2018) 10:365. 10.3390/nu10030365 29562591 PMC5872783

[21] Tronchon V Vialard F El Sirkasi M Dechaud H Rollet J Albert M. Tumor necrosis factor-alpha−308 polymorphism in infertile men with altered sperm production or motility. Hum Reprod. (2008) 23:2858–66. 10.1093/humrep/den277 18689851

[22] Eslamian G Amirjannati N Rashidkhani B Sadeghi MR Baghestani AR Hekmatdoost A. Adherence to the western pattern is potentially an unfavorable indicator of asthenozoospermia risk: a case-control study. J Am Coll Nutr. (2016) 35:50–8. 10.1080/07315724.2014.936983 25764357

[23] Organization WH. WHO Laboratory Manual for the Examination and Processing of Human Semen. 5th ed. Geneva: World Health Organization. (2010).

[24] Eslamian G Amirjannati N Rashidkhani B Sadeghi MR Baghestani AR Hekmatdoost A. Dietary fatty acid intakes and asthenozoospermia: a case-control study. Fertil Steril. (2015) 103:190–8. 10.1016/j.fertnstert.2014.10.010 25456794

[25] Du H Bennett D Li L Whitlock G Guo Y Collins R. Physical activity and sedentary leisure time and their associations with BMI, waist circumference, and percentage body fat in 05 million adults: the China Kadoorie Biobank study. Am J Clin Nutr. (2013) 97:487–96. 10.3945/ajcn.112.046854 23364014 PMC4345799

[26] Ainsworth BE Haskell WL Herrmann SD Meckes N Bassett DR Jr Tudor-Locke C. 2011 Compendium of Physical Activities: a second update of codes and MET values. Med Sci Sports Exerc. (2011) 43:1575–81. 10.1249/MSS.0b013e31821ece12 21681120

[27] Stierman B Ogden CL Yanovski JA Martin CB Sarafrazi N Hales CM. Changes in adiposity among children and adolescents in the United States, 1999-2006 to 2011-2018. Am J Clin Nutr. (2021) 114:1495–504. 10.1093/ajcn/nqab237 34291279 PMC8645193

[28] Zhou BF. Cooperative Meta-Analysis Group of the Working Group on Obesity in China. Predictive values of body mass index and waist circumference for risk factors of certain related diseases in Chinese adults–study on optimal cut-off points of body mass index and waist circumference in Chinese adults. Biomed Environ Sci. (2002) 15:83–96. 12046553

[29] Zhang HH Zhao YH. Ambient air pollution exposure during pregnancy and gestational diabetes mellitus in Shenyang, China: a prospective cohort study. Environ Sci Pollut Res Int. (2021) 28:7806–14. 10.1007/s11356-020-11143-x 33037545

[30] Zhang HH Xia Y Chang Q Gao S Zhao YH. Dietary patterns and associations between air pollution and gestational diabetes mellitus. Environ Int. (2021) 147:106347. 10.1016/j.envint.2020.106347 33385926

[31] Wang XB Wu QJ Guo RH Leng X Du Q Zhao YH. Dairy product consumption and oligo-astheno-teratozoospermia risk: a hospital-based case-control study in china. Front Nutr. (2021) 8:742375. 10.3389/fnut.2021.742375 34993218 PMC8724031

[32] Cui Q Xia Y Liu Y Sun Y Ye K Li W. Validity and reproducibility of a food frequency questionnaire for assessing dietary intake among residents of northeast china: northeast cohort study of China. Br J Nutr. (2022) 1−40. 10.1017/S0007114522002318 35912695

[33] Yang Y Wang G Pan X. China Food Composition. 2nd edn. Beijing: Peking University Medical Press. (2009).

[34] Wen ZY Liu C Liu FH Wei YF Xu HL Wang R. Association between pre-diagnostic dietary pattern and survival of ovarian cancer: evidence from a prospective cohort study. Clin Nutr. (2022) 41:452–9. 10.1016/j.clnu.2021.12.033 35007814

[35] Bonetto C Pace D Bodini L Colombi M Van Bortel T Lasalvia A. Development and psychometric validation of new questionnaires assessing experienced discrimination and internalised stigma among people with COVID-19. Epidemiol Psychiatr Sci. (2022) 31:e37. 10.1017/S204579602200021X 35616053 PMC9158394

[36] Xia Y Zhang Q Liu L Meng G Wu H Bao X. Intermediary effect of inflammation on the association between dietary patterns and non-alcoholic fatty liver disease. Nutrition. (2020) 71:110562. 10.1016/j.nut.2019.110562 31809956

[37] Poti JM Braga B Qin B. Ultra-processed food intake and obesity: what really matters for health-processing or nutrient content? Curr Obes Rep. (2017) 6:420–31. 10.1007/s13679-017-0285-4 29071481 PMC5787353

[38] Cui Q Wang HH Wu QJ Wang XB Guo RH Leng X. Diet quality scores and asthenoteratozoospermia risk: finding from a hospital-based case-control study in China. Front Nutr. (2022) 9:859143. 10.3389/fnut.2022.859143 35479758 PMC9036176

[39] Eslamian G Amirjannati N Rashidkhani B Sadeghi MR Hekmatdoost A. Intake of food groups and idiopathic asthenozoospermia: a case-control study. Hum Reprod. (2012) 27:3328–36. 10.1093/humrep/des311 22940769

[40] Martinez Steele E Popkin BM Swinburn B Monteiro CA. The share of ultra-processed foods and the overall nutritional quality of diets in the US: evidence from a nationally representative cross-sectional study. Popul Health Metr. (2017) 15:6. 10.1186/s12963-017-0119-3 28193285 PMC5307821

[41] Louzada M Ricardo CZ Steele EM Levy RB Cannon G Monteiro CA. The share of ultra-processed foods determines the overall nutritional quality of diets in Brazil. Public Health Nutr. (2018) 21:94–102. 10.1017/S1368980017001434 28714425 PMC10260839

[42] Beslay M Srour B Méjean C Allès B Fiolet T Debras C. Ultra-processed food intake in association with BMI change and risk of overweight and obesity: a prospective analysis of the French NutriNet-Santé cohort. PLoS Med. (2020) 17:e1003256. 10.1371/journal.pmed.1003256 32853224 PMC7451582

[43] Mendonca RD Pimenta AM Gea A de la Fuente-Arrillaga C Martinez-Gonzalez MA Lopes AC. Ultraprocessed food consumption and risk of overweight and obesity: the University of Navarra Follow-Up (SUN) cohort study. Am J Clin Nutr. (2016) 104:1433–40. 10.3945/ajcn.116.135004 27733404

[44] Belloc S Cohen-Bacrie M Amar E Izard V Benkhalifa M Dalléac A. High body mass index has a deleterious effect on semen parameters except morphology: results from a large cohort study. Fertil Steril. (2014) 102:1268–73. 10.1016/j.fertnstert.2014.07.1212 25225071

[45] Liu Y Guo Y Song N Fan Y Li K Teng X. Proteomic pattern changes associated with obesity-induced asthenozoospermia. Andrology. (2015) 3:247–59. 10.1111/andr.289 25293813

[46] Chassaing B Van de Wiele T De Bodt J Marzorati M Gewirtz AT. Dietary emulsifiers directly alter human microbiota composition and gene expression ex vivo potentiating intestinal inflammation. Gut. (2017) 66:1414–27. 10.1136/gutjnl-2016-313099 28325746 PMC5940336

[47] Chassaing B Koren O Goodrich JK Poole AC Srinivasan S Ley RE. Dietary emulsifiers impact the mouse gut microbiota promoting colitis and metabolic syndrome. Nature. (2015) 519:92–6. 10.1038/nature14232 25731162 PMC4910713

[48] Lundy SD Sangwan N Parekh NV Selvam MKP Gupta S McCaffrey P. Functional and taxonomic dysbiosis of the gut, urine, and semen microbiomes in male infertility. Eur Urol. (2021) 79:826–36. 10.1016/j.eururo.2021.01.014 33573862

[49] Ding N Zhang X Zhang XD Jing J Liu SS Mu YP. Impairment of spermatogenesis and sperm motility by the high-fat diet-induced dysbiosis of gut microbes. Gut. (2020) 69:1608–19. 10.1136/gutjnl-2019-319127 31900292 PMC7456731

[50] Wang Y Sun Y Zhao X Yuan R Jiang H Pu X. Downregulation of DJ-1 Fails to Protect Mitochondrial Complex I Subunit NDUFS3 in the Testes and Contributes to the Asthenozoospermia. Mediators Inflamm. (2018) 2018:6136075. 10.1155/2018/6136075 29849492 PMC5903298

[51] Ghandehari-Alavijeh R Zohrabi D Tavalaee M Nasr-Esfahani MH. Association between expression of TNF-α, P53 and HIF1α with asthenozoospermia. Hum Fertil (Camb). (2019) 22:145–51. 10.1080/14647273.2018.1493750 30222022

[52] Serrano SE Braun J Trasande L Dills R Sathyanarayana S. Phthalates and diet: a review of the food monitoring and epidemiology data. Environ Health. (2014) 13:43. 10.1186/1476-069X-13-43 24894065 PMC4050989

[53] Buckley JP Kim H Wong E Rebholz CM. Ultra-processed food consumption and exposure to phthalates and bisphenols in the US National Health and Nutrition Examination Survey, 2013-2014. Environ Int. (2019) 131:105057. 10.1016/j.envint.2019.105057 31398592 PMC6728187

[54] Barakat R Lin PP Rattan S Brehm E Canisso IF Abosalum ME. Prenatal exposure to DEHP induces premature reproductive senescence in male mice. Toxicol Sci. (2017) 156:96–108. 10.1093/toxsci/kfw248 28082598 PMC6075616

[55] D'Avanzo B La Vecchia C Katsouyanni K Negri E Trichopoulos D. An assessment, and reproducibility of food frequency data provided by hospital controls. Eur J Cancer Prev. (1997) 6:288–93. 10.1097/00008469-199706000-00006 9306076

[56] D' Avanzo B La Vecchia C Katsouyanni K Negri E Trichopoulos D. Reliability of information on cigarette smoking and beverage consumption provided by hospital controls. Epidemiology. (1996) 7:312–5. 10.1097/00001648-199605000-00018 8728449

[57] Wang W Tu C Nie H Meng L Li Y Yuan S. Biallelic mutations in CFAP65 lead to severe asthenoteratospermia due to acrosome hypoplasia and flagellum malformations. J Med Genet. (2019) 56:750–7. 10.1136/jmedgenet-2019-106031 31413122 PMC6860412

[58] Zhou N Cui Z Yang S Han X Chen G Zhou Z. Air pollution and decreased semen quality: a comparative study of Chongqing urban and rural areas. Environ Pollut. (2014) 187:145–52. 10.1016/j.envpol.2013.12.030 24491300

