# Supplementary material for: Intake of ultra-processed foods and asthenozoospermia odds: A hospital-based case-control study
Source: Front Nutr. 2022 Oct 20;9:941745. doi: 10.3389/fnut.2022.941745 (PMC9630735; doi:10.3389/fnut.2022.941745)
Supplement: Supplementary file 1 [file Data_Sheet_1.docx]

**Supplemental Table 1.** STROBE Statement—Checklist of items that should be included in reports of ***case-control studies***

|  | | Item No | Recommendation | | Page No |
| --- | --- | --- | --- | --- | --- |
| **Title and abstract** | | 1 | (*a*) Indicate the study’s design with a commonly used term in the title or the abstract | | 1 |
|  |  |  | (*b*) Provide in the abstract an informative and balanced summary of what was done and what was found | | 1 |
| Introduction | | | | | |
| Background/rationale | | 2 | Explain the scientific background and rationale for the investigation being reported | | 2 |
| Objectives | | 3 | State specific objectives, including any prespecified hypotheses | | 2 |
| Methods | | | | | |
| Study design | | 4 | Present key elements of study design early in the paper | | 2 |
| Setting | | 5 | Describe the setting, locations, and relevant dates, including periods of recruitment, exposure, follow-up, and data collection | | 2 |
| Participants | | 6 | (*a*) Give the eligibility criteria, and the sources and methods of case ascertainment and control selection. Give the rationale for the choice of cases and controls | | 2 |
|  |  |  | (*b*) For matched studies, give matching criteria and the number of controls per case | | NA |
| Variables | | 7 | Clearly define all outcomes, exposures, predictors, potential confounders, and effect modifiers. Give diagnostic criteria, if applicable | | 3-4 |
| Data sources/ measurement | | 8* | For each variable of interest, give sources of data and details of methods of assessment (measurement). Describe comparability of assessment methods if there is more than one group | | 3-4 |
| Bias | | 9 | Describe any efforts to address potential sources of bias | | 4 |
| Study size | | 10 | Explain how the study size was arrived at | | 4 |
| Quantitative variables | | 11 | Explain how quantitative variables were handled in the analyses. If applicable, describe which groupings were chosen and why | | 4 |
| Statistical methods | | 12 | (*a*) Describe all statistical methods, including those used to control for confounding | | 4 |
|  |  |  | (*b*) Describe any methods used to examine subgroups and interactions | | 4 |
|  |  |  | (*c*) Explain how missing data were addressed | | 2 |
|  |  |  | (*d*) If applicable, explain how matching of cases and controls was addressed | | NA |
|  |  |  | (*e*) Describe any sensitivity analyses | | 4 |
| Results | | | | | |
| Participants | | 13* | (a) Report numbers of individuals at each stage of study—eg numbers potentially eligible, examined for eligibility, confirmed eligible, included in the study, completing follow-up, and analysed | | Figure 1 |
|  |  |  | (b) Give reasons for non-participation at each stage | | Figure 1 |
|  |  |  | (c) Consider use of a flow diagram | | Figure 1 |
| Descriptive data | | 14* | (a) Give characteristics of study participants (eg demographic, clinical, social) and information on exposures and potential confounders | | Table 1 |
|  |  |  | (b) Indicate number of participants with missing data for each variable of interest | | Figure 1 |
| Outcome data | | 15* | Report numbers in each exposure category, or summary measures of exposure | | Table 2 |
| Main results | 16 | | | (*a*) Give unadjusted estimates and, if applicable, confounder-adjusted estimates and their precision (eg, 95% confidence interval). Make clear which confounders were adjusted for and why they were included | Table 2 |
|  |  |  |  | (*b*) Report category boundaries when continuous variables were categorized | NA |
|  |  |  |  | (*c*) If relevant, consider translating estimates of relative risk into absolute risk for a meaningful time period | NA |
| Other analyses | 17 | | | Report other analyses done—eg analyses of subgroups and interactions, and sensitivity analyses | 4 |
| Discussion | | | | | |
| Key results | 18 | | | Summarise key results with reference to study objectives | 5 |
| Limitations | 19 | | | Discuss limitations of the study, taking into account sources of potential bias or imprecision. Discuss both direction and magnitude of any potential bias | 7 |
| Interpretation | 20 | | | Give a cautious overall interpretation of results considering objectives, limitations, multiplicity of analyses, results from similar studies, and other relevant evidence | 7 |
| Generalisability | 21 | | | Discuss the generalisability (external validity) of the study results | 7 |
| Other information | | | | | |
| Funding | 22 | | | Give the source of funding and the role of the funders for the present study and, if applicable, for the original study on which the present article is based | 7 |

NA, not applicable.

*Give information separately for cases and controls.

**Note:** An Explanation and Elaboration article discusses each checklist item and gives methodological background and published examples of transparent reporting. The STROBE checklist is best used in conjunction with this article (freely available on the Web sites of PLoS Medicine at http://www.plosmedicine.org/, Annals of Internal Medicine at http://www.annals.org/, and Epidemiology at http://www.epidem.com/). Information on the STROBE Initiative is available at http://www.strobe-statement.org.

**Supplemental Table 2. Factor loadings scores of food items of dietary patterns.**

| **Food items** | **Vegetables pattern** | **Fruit pattern** | **Fish**  **pattern** | **Processed foods pattern** | **Tea**  **pattern** |
| --- | --- | --- | --- | --- | --- |
| radish (expect for carrot) | **0.67^*^** | 0.05 | 0.27 | 0.02 | 0.10 |
| cauliflower | **0.64** | 0.11 | 0.29 | -0.09 | 0.10 |
| carrot | **0.64** | 0.08 | 0.15 | 0.01 | 0.06 |
| pakchoi | **0.64** | 0.17 | 0.27 | 0.01 | -0.18 |
| agaric | **0.63** | 0.09 | 0.08 | 0.11 | 0.13 |
| cabbage | **0.63** | 0.12 | 0.26 | 0.00 | -0.12 |
| rape | **0.62** | 0.12 | 0.37 | -0.03 | -0.16 |
| bell peppers | **0.61** | 0.04 | 0.04 | 0.10 | 0.06 |
| Chinese cabbage | **0.60** | 0.11 | 0.2 | 0.01 | -0.17 |
| garlic sprout | **0.59** | 0.14 | 0.06 | 0.18 | 0.13 |
| onion | **0.58** | 0.08 | 0.01 | 0.15 | 0.15 |
| celery | **0.58** | 0.07 | 0.19 | -0.04 | 0.03 |
| eggplant | **0.58** | 0.11 | -0.08 | 0.06 | -0.09 |
| mushroom | **0.56** | 0.13 | 0.15 | 0.15 | 0.07 |
| cucumber | **0.56** | 0.19 | -0.03 | 0.06 | -0.1 |
| spinach | **0.56** | 0.14 | 0.34 | 0.03 | -0.18 |
| bean sprout | **0.54** | 0.05 | 0.1 | 0.21 | 0.05 |
| mung bean | **0.54** | 0.10 | -0.12 | 0.13 | -0.04 |
| Chinese watermelon | **0.53** | 0.04 | 0.36 | -0.09 | 0.08 |
| tomato | **0.53** | 0.16 | 0.14 | 0.07 | -0.13 |
| pumpkin | **0.52** | 0.10 | 0.43 | -0.12 | 0.09 |
| broccoli | **0.50** | 0.13 | 0.31 | -0.09 | 0.08 |
| leek | **0.49** | 0.11 | 0.06 | 0.12 | 0.07 |
| other types of beans | **0.44** | 0.09 | 0.21 | 0.11 | 0.16 |
| hot pepper | **0.43** | 0.03 | -0.11 | 0.24 | 0.09 |
| sea plant | **0.41** | 0.07 | 0.30 | 0.15 | 0.14 |
| tofu | **0.39** | 0.07 | 0.07 | 0.26 | 0.02 |
| garlic | **0.38** | 0.07 | -0.07 | 0.32 | -0.04 |
| green Chinese onion | **0.38** | 0.14 | -0.15 | 0.29 | -0.06 |
| bean | **0.37** | 0.09 | 0.28 | 0.02 | 0.09 |
| potatoes | **0.33** | 0.03 | -0.08 | 0.19 | -0.07 |
| sweet potatoes | **0.32** | 0.12 | 0.28 | 0.06 | -0.08 |
| lotus root | **0.31** | 0.13 | 0.33 | 0.07 | 0.05 |
| soybean milk without sugar | **0.31** | 0.10 | 0.16 | 0.15 | 0.01 |
| pineapple | 0.07 | **0.76** | 0.12 | 0.01 | 0.08 |
| orange | 0.14 | **0.72** | 0.11 | 0.02 | 0.07 |
| peach | 0.05 | **0.71** | 0.07 | -0.01 | -0.01 |
| grape | 0.12 | **0.69** | 0.04 | 0.05 | 0.03 |
| pear | 0.12 | **0.67** | 0.14 | 0.01 | -0.04 |
| watermelon | 0.05 | **0.67** | -0.02 | 0.07 | -0.01 |
| banana | 0.19 | **0.59** | 0.10 | -0.01 | 0.00 |
| strawberry | 0.11 | **0.58** | 0.16 | 0.02 | 0.17 |
| kiwi fruit | 0.17 | **0.57** | 0.23 | -0.06 | 0.15 |
| apple | 0.19 | **0.52** | 0.10 | -0.02 | -0.04 |
| nut | 0.14 | **0.45** | 0.21 | 0.11 | 0.11 |
| other kinds of fruits | 0.09 | **0.39** | 0.15 | 0.01 | 0.11 |
| peanut | 0.19 | **0.37** | 0.04 | 0.19 | 0.02 |
| Chinese sauerkraut | 0.19 | **0.35** | -0.03 | 0.20 | 0.10 |
| melon | 0.13 | **0.33** | -0.03 | 0.25 | -0.06 |
| yam | 0.28 | 0.11 | **0.40** | 0.03 | -0.02 |
| fresh or pasteurized milk | 0.16 | 0.10 | **0.40** | -0.03 | 0.01 |
| fried fish | 0.12 | 0.10 | **0.40** | 0.23 | 0.07 |
| saltwater fish | 0.12 | 0.06 | **0.37** | 0.07 | 0.14 |
| skim milk | 0.15 | 0.05 | **0.37** | -0.07 | -0.01 |
| animal liver | 0.02 | 0.04 | **0.37** | 0.37 | 0.04 |
| coarse cereals | 0.29 | 0.05 | **0.34** | -0.11 | -0.03 |
| shrimp | 0.00 | 0.05 | **0.34** | 0.17 | 0.12 |
| other pickles | 0.18 | 0.06 | -0.11 | **0.47** | -0.05 |
| dried vegetable | 0.21 | 0.04 | 0.16 | **0.40** | 0.01 |
| pickles | 0.20 | 0.05 | 0.14 | **0.40** | -0.05 |
| salted meat | 0.17 | 0.11 | 0.02 | **0.40** | -0.06 |
| animal offal (except for animal liver) | -0.09 | 0.06 | 0.29 | **0.39** | 0.04 |
| animal blood | 0.02 | 0.04 | 0.24 | **0.36** | -0.02 |
| beer | -0.06 | -0.10 | -0.01 | **0.36** | 0.08 |
| preserved bean curd | 0.15 | 0.07 | 0.12 | **0.33** | 0.09 |
| salted egg | 0.23 | 0.10 | -0.07 | **0.33** | -0.04 |
| red tea | 0.05 | 0.08 | 0.03 | 0.07 | **0.60** |
| green tea | 0.02 | 0.11 | 0.05 | 0.06 | **0.60** |
| Oolong tea | 0.03 | 0.10 | 0.09 | 0.08 | **0.53** |
| Pu'er tea | 0.1 | 0.05 | 0.08 | -0.03 | **0.47** |
| jasmine tea | -0.01 | 0.08 | 0.06 | -0.01 | **0.43** |
| strong liquor | -0.01 | -0.08 | 0.04 | 0.29 | 0.19 |
| wine | 0.01 | 0.04 | 0.22 | 0.09 | 0.13 |
| honey | 0.11 | 0.15 | 0.19 | 0.01 | 0.13 |
| coffee | -0.01 | 0.00 | 0.12 | -0.03 | 0.12 |
| meat | 0.02 | -0.01 | 0.04 | 0.17 | 0.11 |
| low-alcohol liquor | 0.00 | -0.06 | 0.00 | 0.23 | 0.09 |
| soy | 0.24 | 0.07 | 0.14 | 0.03 | 0.08 |
| ginger | 0.19 | 0.04 | 0.02 | 0.1 | 0.07 |
| freshwater fish | 0.14 | 0.02 | 0.26 | 0.12 | 0.05 |
| yellow rice wine | -0.01 | -0.01 | 0.08 | 0.06 | 0.04 |
| freshly made unpackaged cheeses | 0.04 | 0.07 | 0.25 | 0.05 | 0.04 |
| yoghourt | 0.02 | 0.13 | 0.29 | 0.00 | 0.03 |
| jujube (red date) | 0.22 | 0.15 | 0.24 | -0.01 | 0.02 |
| poultry | 0.08 | 0.07 | 0.27 | 0.21 | 0.01 |
| eggs | 0.25 | 0.03 | 0.29 | 0.04 | 0.01 |
| steamed bread | 0.20 | 0.03 | 0.21 | 0.04 | 0.00 |
| miscellaneous sauce noodles | 0.05 | 0.10 | 0.18 | 0.17 | -0.02 |
| wonton | 0.05 | 0.02 | 0.26 | 0.09 | -0.03 |
| fried egg | 0.26 | 0.07 | 0.20 | 0.16 | -0.04 |
| deep-fried dough sticks | 0.05 | 0.09 | 0.23 | 0.14 | -0.04 |
| pork skin | 0.07 | 0.07 | 0.06 | 0.24 | -0.07 |
| rice | -0.01 | 0.04 | -0.22 | 0.04 | -0.07 |
| congee | 0.21 | 0.06 | 0.21 | 0.04 | -0.08 |
| steamed stuffed bun and dumpling | 0.12 | 0.02 | 0.17 | 0.13 | -0.13 |
| vermicelli and sheet jelly | 0.08 | 0.04 | 0.14 | 0.25 | -0.15 |

**^*^**Factor loadings represent the relative contribution of each food item to dietary patterns. The five groups with highest factor loadings in each dietary pattern had been shown in bold.

**Supplemental Table 3.** Food items in the food frequency questionnaire according to the NOVA classification system.

| **Group** ^a^ | **Food items** |
| --- | --- |
| Unprocessed or minimally processed foods | Rice, steamed bread, congee, coarse cereals, vermicelli, sheet jelly, fresh or pasteurized milk, skim milk, yoghourt, poultry, meat, animal liver, animal offal (except for animal liver), pork skin, animal blood, egg, fish and seafood (shrimp, saltwater fish, freshwater fish), starchy roots and tubers (potatoes, sweet potatoes and yam), fresh vegetables (Chinese cabbage, pakchoi, cabbage, rape, spinach, tomato, pumpkin, cucumber, Chinese watermelon, celery, eggplant, broccoli, cauliflower, carrot, radish, bell peppers, hot pepper, agaric, mushroom, leek, shallot, onion, garlic sprout, sea plant, lotus root, jujube (red date), green Chinese onion, garlic, and ginger), legumes (soy, bean, mung bean, bean sprout, other types of beans, soybean milk without sugar, tofu), fresh fruits (apple, pear, orange, banana, grape, watermelon, peach, pineapple, strawberry, kiwi fruit, and other kinds of fruits), seeds (melon seed and peanut) and nut, green tea, red tea, oolong tea, Pu'er tea, jasmine tea, and coffee |
| Processed culinary ingredients | Honey |
| Processed foods | Miscellaneous sauce noodles, wonton, dumpling, steamed stuffed bun, deep-fried dough sticks, freshly made unpackaged cheeses, pickle, dried vegetable, Chinese sauerkraut, fried egg, salted egg, fried fish, salted meat, preserved bean curd |
| Ultra-processed foods | Instant noodles, bread, sausages, preserved egg, western-style pastry or cakes, Chinese pastries or cakes, cookies, sweet snacks or candies or jams, chocolate, ice cream, sesame/soybean paste, vegetable/fruit drinks, and soft drinks |

^a^ Alcoholic drinks are not classifiable by NOVA

**Supplemental Table 4.** Adjusted odds ratio (95% confidence interval) of absolute amount of ultra-processed foods intake and asthenozoospermia.

| **Models** | **Tertiles of UPFs intake** | | | ***P* for trend** |
| --- | --- | --- | --- | --- |
|  | T1 | T2 | T3 |  |
| Range (g/day) | < 71.81 | 71.81 to ≤ 162.23 | > 162.23 |  |
| Case/Control | 155/194 | 191/193 | 203/194 |  |
| Model 1 | 1.00 (Ref) | 1.35 (1.00, 1.81) | 1.40 (1.01, 1.95) | < 0.05 |
| Model 2 | 1.00 (Ref) | 1.38 (1.02, 1.88) | 1.52 (1.08, 2.13) | < 0.05 |
| Model 3 | 1.00 (Ref) | 1.39 (1.03, 1.89) | 1.53 (1.08, 2.16) | < 0.05 |

T, tertiles; UPF, ultra-processed foods.

Model 1: adjusted for age, body mass index, and total energy intake.

Model 2: adjusted for age, annual family income, educational level, body mass index, smoking status, drinking status, abstinence time, physical activity, total energy intake, and dietary change status.

Model 3: adjusted for age, annual family income, educational level, body mass index, smoking status, drinking status, abstinence time, physical activity, total energy intake, dietary change status, and dietary patterns except UPF foods (vegetables pattern, fruit pattern, fish pattern, pickle pattern, and tea pattern).

**Supplemental Table 5.** Adjusted odds ratio (95% confidence interval) for asthenozoospermia according to unprocessed or minimally processed foods and processed foods intake.

| **Models** | **Tertiles of intake** | | | ***P* for trend** |
| --- | --- | --- | --- | --- |
|  | **T1** | **T2** | **T3** |  |
| **Unprocessed or minimally processed foods** |  |  |  |  |
| Range (% total energy) | < 68.85 | 68.85 to ≤ 78.76 | > 78.76 |  |
| Case/Control | 233/194 | 193/193 | 123/194 |  |
| Model 1 | 1.00 (Ref) | 0.81 (0.61, 1.07) | 0.49 (0.36, 0.67) | < 0.05 |
| Model 2 | 1.00 (Ref) | 0.72 (0.54, 0.97) | 0.40 (0.29, 0.56) | < 0.05 |
| Model 3^a^ | 1.00 (Ref) | 0.61 (0.45, 0.83) | 0.32 (0.22, 0.46) | < 0.05 |
| **Processed foods** |  |  |  |  |
| Range (% total energy) | < 6.74 | 6.74 to ≤ 10.43 | > 10.43 |  |
| Case/Control | 149/194 | 177/193 | 223/194 |  |
| Model 1 | 1.00 (Ref) | 1.14 (0.84, 1.53) | 1.47 (1.10, 1.98) | < 0.05 |
| Model 2 | 1.00 (Ref) | 1.15 (0.85, 1.56) | 1.48 (1.10, 2.00) | < 0.05 |
| Model 3^b^ | 1.00 (Ref) | 1.15 (0.85, 1.56) | 1.48 (1.10, 2.01) | 0.08 |

T, tertile; Ref, reference.

Model 1: adjusted for age, body mass index, and total energy intake.

Model 2: adjusted for age, annual family income, educational level, body mass index, cigarette smoking, alcohol drinking, abstinence time, physical activity, total energy intake, and dietary change status.

Model 3^a^: adjusted for age, annual family income, educational level, body mass index, cigarette smoking, alcohol drinking, abstinence time, physical activity, total energy intake, dietary change status, and dietary patterns except unprocessed or minimally processed foods (ultra-processed foods pattern, processed foods pattern, drinking pattern).

Model 3^b^: adjusted for age, annual family income, educational level, body mass index, cigarette smoking, alcohol drinking, abstinence time, physical activity, total energy intake, dietary change status, and dietary patterns except unprocessed or minimally processed foods (vegetables pattern, fruit pattern, ultra-processed foods pattern, ethnic pattern, and tea pattern).
